# Editorial: Transcriptional Regulation as a Key Player in Cancer Cells Drug Resistance

**DOI:** 10.3389/fonc.2021.764506

**Published:** 2021-10-26

**Authors:** Eva Martinez-Balibrea, Yari Ciribilli

**Affiliations:** ^1^ Germans Trias i Pujol Research Institute (IGTP), ProCURE program, Catalan Institute of Oncology, Badalona, Spain; ^2^ Laboratory of Molecular Cancer Research, Department of Cellular, Computational and Integrative Biology (CIBIO), University of Trento, Trento, Italy

**Keywords:** resistance, biomarkers, EMT – epithelial to mesenchymal transition, transcription factors, chromatin regulators, chemotherapy, targeted therapy, immunotherapy

In cancer, genetic and epigenetic alterations lead to dysregulated transcriptional programs making cancer cells highly dependent on certain regulators of gene expression. Such regulators, as transcription factors (TFs), are culprits of almost if not all the hallmarks of cancer (1). For instance, SLUG and SNAIL are involved in the epithelial-to-mesenchymal transition (EMT) (2), while STAT3 is one of the main effectors downstream the EGFR pathway (3). More recently, non-coding RNAs (miRNAs, LncRNAs, etc) have been revealed as important transcription regulators whose alteration is involved in several mechanisms leading to carcinogenesis, tumor progression, or therapy resistance (4–8). The latest is an critical phenomenon causing treatment failures and reducing patients’ survival. Most chemotherapy drugs induce DNA damage that ultimately promotes cancer cells’ death. Alterations in transcription factors or chromatin regulators have been shown to affect the ability of a tumor for responding to such damage and, in turn, to facilitate its survival making it resistant to the treatment (9). Several examples exist but a well-known case is that involving the Nuclear factor kappa B (NF-κB) protein. Activation of the NF-κB pathway has been involved in the resistance to multiple chemotherapy drugs including Fluoropyrimidines (10), Taxanes (11) or Platin-derivatives (12) in a variety of solid tumors. Similarly to chemotherapy, resistance to targeted therapies is also a major problem in cancer. The reduced sensitivity of cancer cells to this kind of drugs is often related to alterations in the driver oncogene, activation of a critical signaling pathway, and to pro-survival signaling through a different signaling pathway (13). In the last case, transcription factors such as FOXO, RUNX1 and RUNX2 play important roles in mediating resistance to lapatinib, quizartinib or vemurafenib, respectively (14). More recently, immunotherapies based on immune checkpoint blocking have been approved for the treatment of several malignancies including non-small cell lung cancer, melanoma or colorectal cancer with microsatellite instability, among others (15). Although their undoubtable and striking results in some settings, tumors often develop resistance against them. And again, transcription regulators have been shown to be involved in such resistance mechanisms. For instance, MYC drives evasion of CD4+ T cells and decreases expression of PD-L1; STAT1 regulates expression of PD-L1 in melanomas and RUNX1-ETO reduces CD48, leading to decreased NK cell killing (16). Moreover, chromatin-remodeling pathways contribute to cancer cell immune resistance for instance through the control of interferon-stimulated gene expression (17, 18). Finally, but not less important, radiotherapy resistance is oftentimes related to alterations in transcription regulation. HIF-1α is one of the most frequently studied TF related to radiotherapy resistance. HIF-1α promotes glycolysis and the pentose phosphate pathway which in turn increases the antioxidant capacity of tumors, counteracting the oxidative stress caused by irradiation (19). Thus, the importance of transcriptional regulation in the development of anticancer treatment resistance is evident and makes relevant the necessity of developing new strategies to address this problem. In this special issue, we have collected a series of original papers and reviews covering several aspects about the role of transcription alterations in cancer therapeutic resistance.

Three of them demonstrated novel mechanisms of resistance to different sources of standard DNA-damaging chemotherapy such as 5-Fluorouracil, Docetaxel, and Taxanes in general. Specifically, Zhang and collaborators suggested that the inhibition of the GLI1 transcription factor can sensitize colorectal cancer (CRC) patients to the treatment with 5-Fluorouracil (5-FU), one of the most widely used anti-cancer drugs for the management of CRC (20). This effect was based on the non-canonical stimulation through oncogenic BRAF (the constitutively active form determined by the common mutation V600E) of the GLI1 transcriptional activity. GLI1, in turn, induced the expression levels of the NBS1 (Nijmegen Breakage Syndrome-1) gene, which favored the DNA-Damage Response, resulting in the enhanced survival of cancer cells to 5-FU. The authors also demonstrated that the pharmacological inhibition of GLI1 with SRI-38832 (a novel GLI1 antagonist) restored the sensitivity to 5-FU through this axis, even more potently than GANT61, a common pan-GLI family inhibitor.

Besides, Huang and co-workers described how prostate cancer cells could acquire resistance to Docetaxel treatment (21). The reduced sensitivity to Docetaxel depended on the increased expression of Estrogen Receptor-related Receptor alpha (ERRα). The authors revealed by Chromatin Immunoprecipitation and Luciferase assays that ERRα was able to directly induce the expression levels of ABCC4, one of the members of the ATP Binding Cassette (ABC) family. These proteins are efflux pumps responsible for the reduced accumulation into the cells of the drug, as shown in this study. This work pinpoints ERRα as a potential target for personalized therapy in patients that do not respond to Docetaxel treatment.

In the third study, Chen and co-authors determined an increased expression of miR-335-5p and let-7c-5p in breast cancer patients resistant to Taxanes (either Paclitaxel or Docetaxel); as expected, elevated levels of these two miRNAs were also associated with a poorer prognosis (22). Mechanistically, using breast cancer cell line-based models (MCF7 and BCap37) resistant to Taxanes, they showed miR-335-5p and let-7c-5p through the inhibition of CXCL9 and, SOCS1 and CCR7, respectively, can both stimulate cell proliferation and reduce apoptosis, resulting in enhanced resistance to Taxanes. This effect can be reversed by the inhibition of these two miRNAs or by the over-expression of CXCL9, SOCS1, or CCR7.

Moreover, two other studies highlighted novel mechanisms of resistance to Receptor Tyrosine Kinase Inhibitors (TKI) such as Gefitinib (EGFR inhibitor) and Sunitinib (a wide-spectrum TKI with PDGFR, VEGFRs, and c-Kit among the target receptors). In the former one, Wei and colleagues analyzed genome-wide the transcriptome of the lung cancer-derived cell line PC9 resistant to Gefitinib. On top of the commonly found EGFR acquired mutation T790M (which renders the drug ineffective), they identified several additional altered pathways which might be exploited therapeutically. Interestingly, among these pathways, they shortlisted 3 drugs, Dasatinib (an SRC, ABL, and c-Kit inhibitor), KPT-185 (a CRM1 inhibitor), and Pluripotin (a dual inhibitor of ERK1 and RasGAP) that could reduce cell growth, induce apoptosis, and block cell migration of Gefitinib-resistant cells. These results can open new avenues to be pursued to overcome the resistance to Gefitinib.

In the latter, Shan and collaborators identified the long non-coding RNA (lncRNA) CCAT1 (Colon Cancer-Associated Transcript-1 (CCAT1) as a mediator of Sunitinib resistance in renal cell carcinoma (RCC) (23). The authors found CCAT1 levels elevated in Sunitinib-resistant ACHN cells coupled with an increased expression of c-Myc and anti-apoptotic proteins BCL-2 and MCL-1. The down-regulation of CCAT1 restored the sensitivity of RCC cells to Sunitinib both *in vitro* and *in vivo*. Interestingly, CCAT1 levels were higher in RCC patients that did not respond to Sunitinib administration.

Furthermore, Zeng and colleagues analyzed the role of miR-381 in cancer development and progression (24). This review provides crucial insights into this miRNA’s functions, given its impact is still debated, even if most reports support the idea to consider miR-381 as a tumor suppressor. Indeed, it is usually found down-regulated in several cancer tissues (with the sole exception of gliomas). They found it particularly relevant for Epithelial-to-Mesenchymal Transition, where miR-381 was demonstrated to target and block the functions of pro-EMT genes such as TWIST1, SOX4, or ZEB1. Interestingly, miR-381 was down-regulated in breast cancer cells resistant to Doxorubicin or Cisplatin, and, therefore, the authors proposed miR-381 as a prognostic factor for cancer aggressiveness.

Lastly, Galeaz and co-authors comprehensively summarized different examples of intrinsic and acquired radioresistance involving transcription factors (25). Specifically, they focused on 4 TFs, STAT3, NF-κB, NRF2, and HIF-1α, whose transcriptional networks were activated in response to the exposure to radiation therapies and can be responsible for a reduced effectiveness of the treatment. Interestingly, most of them converge on the increase in the Cancer Stem Cell subpopulation within the tumor, inducing inflammation, proliferation, and DNA repair, or reducing the oxidative stress-driven by radiations, and, therefore, making it more refractory to the therapy.

Altogether, with this collection, we intend to point out the relevance that transcriptional regulation has in such an important problem as resistance to cancer treatment. Although further investigation is guaranteed, more investments are needed to validate biomarkers and to develop novel therapies. In this sense, most of the factors mentioned above are challenging to target pharmacologically; however, novel strategies such as protein degradation *via* PROTACs or others may offer new opportunities to translate this knowledge into the clinics in the near future.

## Author Contributions

EM-B and YC conceived the editorial, wrote the first draft, and revised and approved the manuscript. All authors contributed to the article and approved the submitted version.

## Funding

Martinez-Balibrea’s lab is supported by grant 2017-SGR-1705 from the Departament d’Innovació, Universitats i Empresa, Generalitat de Catalunya.

## Conflict of Interest

The authors declare that the research was conducted in the absence of any commercial or financial relationships that could be construed as a potential conflict of interest.

## Publisher’s Note

All claims expressed in this article are solely those of the authors and do not necessarily represent those of their affiliated organizations, or those of the publisher, the editors and the reviewers. Any product that may be evaluated in this article, or claim that may be made by its manufacturer, is not guaranteed or endorsed by the publisher.
